# Genetic screen identifies non-mitochondrial proteins involved in the maintenance of mitochondrial homeostasis

**DOI:** 10.17912/micropub.biology.000562

**Published:** 2022-05-11

**Authors:** Stephane Rolland, Barbara Conradt

**Affiliations:** 1 Faculty of Biology, Ludwig-Maximilians-University Munich, 82152 Planegg-Martinsried, Germany; 2 Current Address: Center for Genomic Integrity, Institute for Basic Science (IBS), Ulsan 44919, South Korea; 3 Center for Integrated Protein Science (CIPSM), Ludwig-Maximilians-University Munich, 82152 Planegg-Martinsried, Germany; 4 Current Address: Department of Cell and Developmental Biology, Division of Biosciences, University College London, London WC1E 6AP, United Kingdom

## Abstract

The mitochondrial unfolded protein response (UPR ^mt^ ) is an important stress response that ensures the maintenance of mitochondrial homeostasis in response to various types of cellular stress. We previously described a genetic screen for *Caenorhabditis elegans* genes, which when inactivated cause UPR ^mt^ activation, and reported genes identified that encode mitochondrial proteins. We now report additional genes identified in the screen. Importantly, these include genes that encode non-mitochondrial proteins involved in processes such as the control of gene expression, post-translational modifications, cell signaling and cellular trafficking. Interestingly, we identified several genes that have been proposed to participate in the transfer of lipids between peroxisomes, ER and mitochondria, suggesting that lipid transfer between these organelles is essential for mitochondrial homeostasis. In conclusion, this study shows that the maintenance of mitochondrial homeostasis is not only dependent on mitochondrial processes but also relies on non-mitochondrial processes and pathways. Our results reinforce the notion that mitochondrial function and cellular function are intimately connected.

**Figure 1. Genetic screen identifies additional proteins involved in the maintenance of mitochondrial homeostasis:**
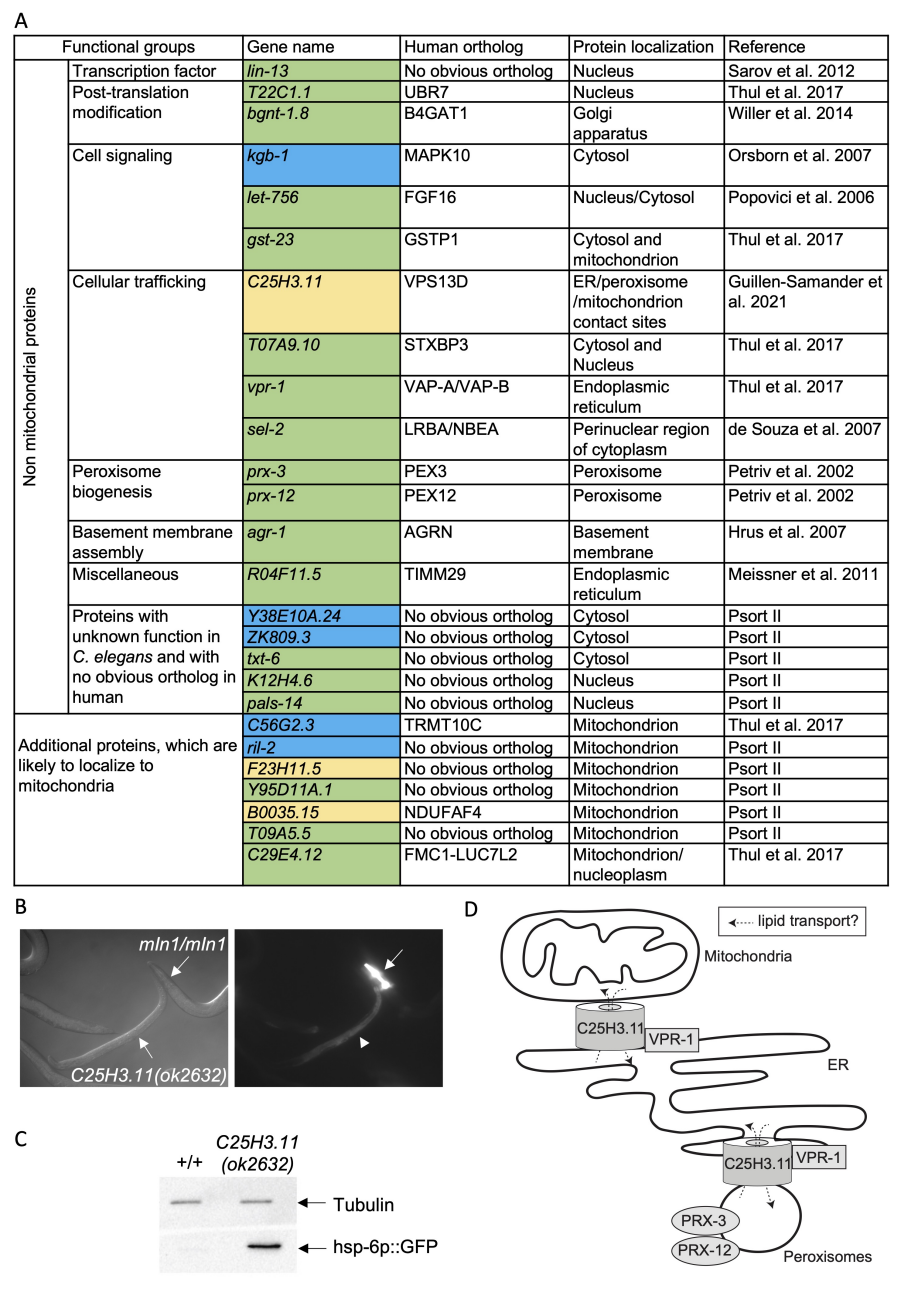
**A.** The candidates identified in the screen are listed by functional groups. When available, the name of the human ortholog as stated on Alliancegenome.org is indicated. Protein localization is indicated and is based on experimental evidence in *C. elegans* or experimental evidence using the human ortholog. In case no experimental evidence was available, the localization was predicted with the PSORT II website (https://psort.hgc.jp/form2.html). Newly identified candidates are indicated in green whereas candidates identified in previous genetic screens are indicated in blue (Runkel et al. 2013, Bennett et al. 2014). Finally, candidates identified in a previous genetic screen for enhancers of *fzo-1* ( *tm1133* lf)-induced UPR ^mt^ are indicated in yellow (Haeussler et al. 2021). **B.** DIC and fluorescence images of animals carrying the *hsp-6p::GFP* ( *zcIs13* ) reporter and either homozygous for the *C25H3.11* loss-of-function allele *ok2632* ( *C25H3.11(ok2632)* ) or homozygous wildtype for *C25H3.11 (mIn1/mIn1).* The balancer *mIn1* carries the *dpy-10* ( *e128* ) mutation and the pharyngeal *myo-2p::GFP* transgene, which is visible in the *mIn1/mIn1* Dumpy animal (indicated by an arrow in the right panel). Induction of the *hsp-6p::GFP* reporter is visible in the intestine of *C25H3.11(ok2632)* homozygous animals (indicated by an arrowhead in the right panel) but not in animals homozygous for the balancer *mIn1* . **C.** Wild-type (+/+) and *C25H3.11* ( *ok2632* ) animals were analyzed by Western analysis using anti-tubulin (loading control) and anti-GFP antibodies. **D.** C25H3.11 VPS13D has been proposed to be a conduit that transfers lipids between ER and mitochondria and between ER and peroxisomes (Guillen-Samander et al. 2021). It localizes to the ER in a VPR-1 VAP-B dependent manner (Guillen-Samander et al. 2021). PRX-3 PEX3 and PRX-12 PEX12 are essential for peroxisome biogenesis and hence peroxisomal beta oxidation (Petriv et al. 2002). Inactivation of any of these genes induces the expression of the *hsp-6p::GFP* reporter and hence triggers UPR ^mt^ , indicating that these proteins are required to maintain mitochondrial homeostasis.

## Description

Mitochondrial UPR (UPR ^mt^ ) is a conserved unfolded protein stress response that is necessary for the maintenance of mitochondrial homeostasis in response to various types of stress (Shpilka et al. 2018). To systematically identify processes that trigger UPR ^mt^ when compromised, we performed a genome-wide RNAi screen in *Caenorhabditis elegans* (Rolland et al. 2019) *.* To that end, we used a reporter construct, *hsp-6p::GFP,* the expression of which is induced in the intestine upon UPR ^mt^ activation (Yoneda et al. 2004). Using this approach, we identified 172 genes that when knocked-down induce the expression of the *hsp-6p::GFP* reporter (and hence activate UPR ^mt^ ) and encode mitochondrial proteins (Rolland et al. 2019). We now report an additional seven genes that fall into this category, which increases the number of genes identified encoding mitochondrial proteins to 179 ( **Figure 1A** ). Furthermore, our screen also led to the identification of 19 genes that induce the expression of the *hsp-6p::GFP* reporter when knocked-down but encode non-mitochondrial proteins ( **Figure 1A** ). In summary, our genetic screen identified in total 179 genes encoding mitochondrial proteins and 19 genes encoding non-mitochondrial proteins, all of which are required for the maintenance of mitochondrial homeostasis. The non-mitochondrial proteins identified have been shown or are predicted to localize to several sub-cellular compartments, including the nucleus, the endoplasmic reticulum (ER), the Golgi apparatus and peroxisomes. This indicates that several cellular pathways including non-mitochondrial pathways participate in the maintenance of mitochondrial homeostasis.

Among the non-mitochondrial proteins identified, we further investigated C25H3.11, the ortholog of mammalian VPS13D (Vacuolar Protein Sorting-associated protein 13D). To confirm this candidate, we analyzed the effect of the *C25H3.11* loss-of-function allele *ok2632* on the expression of the *hsp-6p::GFP* reporter ( *ok2632* is a 1740bp deletion that removes part of exon 3 and 4 and creates a frameshift; it therefore most likely represents a null allele) *.* Since animals homozygous for *ok2632* are sterile, we maintained it over the balancer *mIn1* . Microscopy analysis revealed that the progeny of the balanced strain that is homozygous for *ok2632* ( *C25H3.11* ( *ok2632)* ) exhibits intestinal *hsp-6p::GFP* expression in contrast to animals homozygous for the balancer *(mIn1/mIn1)* **(Figure 1B)** . We confirmed this result by Western analysis ( **Figure 1C** ). Therefore, we conclude that the loss of *C25H3.11* VPS13D induces UPR ^mt^ .

VPS13D was shown to mediate contact sites between mitochondria, ER and peroxisomes (Guillen-Samander et al. 2021). Specifically, VPS13D has been proposed to provide a lipid conduit between peroxisomes and ER as well as between ER and mitochondria. Interestingly, localization of VPS13D to the ER requires the protein VAP-B, the ortholog of another candidate identified in our screen, VPR-1(Guillen-Samander et al. 2021). Inactivation of these genes could affect mitochondrial metabolism by disrupting lipid transport between these organelles. Consistent with this idea, we identified other candidates such as *prx-3* *PEX3* or *prx-12* *PEX12* , which are essential for peroxisome biogenesis and likely cause defects in lipid homeostasis when inactivated. In summary, we propose that the transfer of lipids between mitochondria, ER and peroxisomes is required to maintain mitochondrial homeostasis ( **Figure 1D** ).

We previously showed that by causing a reduction in mitochondrial membrane potential (which is sensed by the UPR ^mt^ transcription factor ATFS-1), the impairment of most but not all mitochondrial processes activates UPR ^mt^ (Rolland et al. 2019). Our new results reveal that non-mitochondrial processes can also activate UPR ^mt^ when compromised. We propose that impairment of these processes might affect mitochondrial membrane potential indirectly through, for example, defects in lipid transfer into and out of mitochondria, thereby inducing UPR ^mt^ .

## Methods

The genetic screen was performed as previously described (Rolland et al. 2019). Western analysis was performed as previously described (Rolland et al. 2019) with the following modifications. Thirty *myo-2p::GFP* negative L4 larvae of MD3815 ( *C25H3.11* ( *ok2632* ) / *mIn1* [ *mIs14* [ *myo-2p::GFP* ] *dpy-10* ( *e128* )] II ; *zcIs13* [ *hsp-6p::GFP* + *lin-15(+)* ] V) ( *C25H3.11* ( *ok2632* )) or thirty L4 larvae of SJ4100 ( *zcIs13* [ *hsp-6p::GFP* + *lin-15(+)* ] V) (+/+) were lysed in Laemmli buffer and analyzed by SDS-PAGE and Western blotting using a monoclonal anti-Tubulin antibody (1:10000; Sigma T6199) and polyclonal anti-GFP antibody (1:6000; Abcam ab290). We used horseradish peroxidase conjugated goat anti-mouse antibodies (BioRad #1706516) at 1:60000 for the anti-Tubulin and a horseradish peroxidase conjugated goat anti-rabbit (BioRad #1706515) at 1:30000 for the anti-GFP. Western was developed using ECL (Amersham #RPN2106) and images were acquired using the ChemiDoc XRS+ System (Bio-Rad).

## Reagents

The Ahringer RNAi library (Kamath et al. 2003) and the *C. elegans* SJ4100 strain ( *zcIs13* [ *hsp-6p::GFP* + *lin-15(+)* ] V) (Yoneda et al. 2004) were used for the genetic screen. The strain VC1998 ( *C25H3.11* ( *ok2632* ) / *mIn1* [ *mIs14* [ *myo-2p::GFP* ] *dpy-10* ( *e128* )] II) was crossed with the SJ4100 strain to generate the strain MD3815 *(C25H3.11* ( *ok2632* ) / *mIn1* [ *mIs14* [ *myo-2p::GFP* ] *dpy-10* ( *e128* )] II ; *zcIs13* [ *hsp-6p::GFP* + *lin-15(+)* ] V).

**Table:** 

**Strain:**	**Genotype:**	**Available from:**
SJ4100	*zcIs13* [ *hsp-6p::GFP* + *lin-15(+)* ] V	CGC
VC1998	*C25H3.11* ( *ok2632* )/ *mIn1* [ *mIs14* [ *myo-2p::GFP* ] *dpy-10* ( *e128* )] II	CGC
MD3815	*C25H3.11* ( *ok2632* )/ *mIn1* [ *mIs14* [ *myo-2p::GFP* ] *dpy-10* ( *e128* )] II ; *zcIs13* [ *hsp-6p::GFP* + *lin-15(+)* ] V	This study
